# Diet-Induced Obesity Promotes Liver Metastasis of Pancreatic Ductal Adenocarcinoma via CX3CL1/CX3CR1 Axis

**DOI:** 10.1155/2022/5665964

**Published:** 2022-04-18

**Authors:** Yue Sun, Xiao-Xin Zhang, Shan Huang, Hong Pan, Yan-Zhi Gai, Yao-Qi Zhou, Lei Zhu, Hui-Zhen Nie, Dong-Xue Li

**Affiliations:** ^1^State Key Laboratory of Oncogenes and Related Genes, Shanghai Cancer Institute, Ren Ji Hospital, Shanghai Jiao Tong University of Medicine, Shanghai 200240, China; ^2^Jiangsu Key Laboratory of Medical Science and Laboratory Medicine, School of Medicine, Jiangsu University, Zhenjiang, 212013 Jiangsu, China

## Abstract

Pancreatic ductal adenocarcinoma (PDAC) is one of the most aggressive cancers, and the patients are generally diagnosed with distant metastasis. Liver is one of the preferred organs of distant metastasis, and liver metastasis is the leading cause of death in PDAC. Diet-induced obesity (DIO) is a risk factor for PDAC, and it remains unclear whether and how DIO contributes to liver metastasis of PDAC. In our study, we found that DIO significantly promoted PDAC liver metastasis compared with normal diet (ND) in intrasplenic injection mouse model. RNA-seq analysis for liver metastasis nodules showed that the various chemokines and several chemokine receptors were altered between ND and DIO samples. The expression levels of CX3CL1 and CX3CR1 were significantly upregulated in DIO-induced liver metastasis of PDAC compared to ND. Increased CX3CL1 promoted the recruitment of CX3CR1-expressing pancreatic tumor cells. Taken together, our data demonstrated that DIO promoted PDAC liver metastasis via CX3CL1/CX3CR1 axis.

## 1. Introduction

Pancreatic cancer, as one of the most lethal cancers, has a 5-year survival rate of about 5% and a median survival rate of about 6 months [1, 2]. Nearly 80% of pancreatic cancer is classified as pancreatic ductal adenocarcinoma (PDAC). PDAC has a poor prognosis owing to its aggressive metastatic nature. The majority of PDAC patients develop distant metastases in progression of the disease. Liver is one of the most preferred destinations of distant metastases in PDAC and is the leading cause of death [3, 4]. Obesity is becoming a major human concern health issue. Several studies have addressed that DIO is a risk factor for multiple types of tumors. Emerging evidence indicated that DIO could activate oncogenic KRAS, leading to pancreatic inflammation and tumorigenesis [5, 6]. DIO promoted desmoplasia associated with impaired delivery of chemotherapeutics and accelerated tumor growth in PDAC [7]. A number of reports have investigated the hepatic dysfunction also caused by DIO [8–10]. Although several reports have demonstrated that DIO was involved in the progression of various malignant tumors, the effect of DIO on liver metastasis of PDAC remains unclear. Therefore, it is crucial for developing patients' effective treatment to understand the mechanism of DIO on liver metastasis of PDAC.

Chemokines are chemotactic cytokines, which are generally regarded as mediators of chronic and acute inflammation, cellular interactions, and tropism. Chemokines are classified on the basis of a conserved variations on cysteine motif in the sequence of the proteins. CX3C motif chemokine ligand 1 (CX3CL1), also called fractalkine, is the only member of CX3C chemokine's subfamily. CX3CL1 exists as two forms that are membrane-bound form and soluble form, which have distinct cellular functions [11]. The membrane-bound form is an adhesion molecule for tumor and endothelial cells through transmembrane hydrophobic region. The soluble form is a chemoattractant for cells expressing CX3CR1 receptor, a unique receptor for CX3CL1 [12]. From the previous reports, CX3CL1/CX3CR1 axis was involved in the pathogenesis of various types of tumors, including breast, gastric, lung, colorectal, and pancreatic cancer [13]. For primary tumor progression, CX3CL1/CX3CR1 played an important role in modulating inflammatory responses associated with macrophage survival and monocyte homeostasis [12]. CX3CL1-CX3CR1 interaction prevented liver fibrosis though increased inflammatory cell recruitment and cytokine production [14]. Meanwhile, angiogenesis and neural tropism were accompanied by high expression of CX3CL1 and CX3CR1 [15]. In nutrient-depleted tumor microenvironment, CX3CL1 stimulated hypoxia-inducible factor (HIF)-1*α* expression through the MAPK and PI3K/Akt pathways subsequently enhanced glucose uptake in PDAC [16]. CX3CL1 was also involved in the pathogenesis of different inflammatory conditions [17, 18]. Further analysis demonstrated that CX3CL1 could directly enhance growth of PDAC cells via the activation of the JAK/STAT pathway [19]. For tumor metastasis, emerging evidence suggested that CX3CL1 regulated metastasis of primary breast, lung, kidney, and prostate tumors [20, 21]. CX3CL1-mediated metastasis was not restricted to the spine, also implicated in lymph node involvement [22]. The chemokine CX3CL1 and its receptor CX3CR1 are involved in the pathogenesis of many types of tumors. However, little is known about the specific role of CX3CL1/CX3CR1 axis in liver metastasis of PDAC.

Here, to explain the effect of DIO in PDAC liver metastasis, we established a DIO mouse model with intrasplenic injection. Our study demonstrated that DIO could promote liver metastasis of PDAC. The expression levels of CX3CL1 and CX3CR1 were increased in DIO-induced liver metastasis of PDAC. Furthermore, our data demonstrated that increased expression of CX3CL1 enhanced the recruitment of PDAC cells expressing CX3CR1, which accelerated the progression of liver metastasis of PDAC.

## 2. Materials and Methods

### 2.1. Cell Lines and Cell Culture

Human PDAC cell lines AsPC-1, PANC-1, Capan-1, CFPAC-1, Mia PaCa-2, and Patu8988 and mouse PDAC cell line Panc02 were all preserved in Shanghai Cancer Institute, Ren Ji Hospital, School of Medicine, Shanghai Jiao Tong University. All cell lines have been performed STR identification and excluded endogenic contamination. The cells were cultured at 37°C in a humidified incubator under 5% CO_2_ condition, with indicated medium containing 10% (*v*/*v*) fetal bovine serum (FBS) and 1% (*v*/*v*) antibiotics, according to American Type Culture Collection (ATCC, Manassas, VA) protocols.

### 2.2. Diet-Induced Obesity Mouse Model

C57BL/6J male mice were originally obtained from The Jackson Laboratory. 5-week-old mice were randomly allocated to either a normal diet (ND) group or a diet-induced obesity (DIO) group. A high-fat diet group was named as DIO group. To generate an obese model, 10% of energy was derived from fat in ND group, and 60% of energy was derived from fat for 12 weeks in DIO group. Body weight and food intake of each animal were measured twice per week.

### 2.3. Intrasplenic Injection Model

To evaluate the liver metastasis potency of tumor cells in vivo, Panc02-luciferase cells (2 × 10^6^ cells in 0.02 ml PBS) were injected into the spleen of ND group and DIO group mice after 12 weeks high-fat feeding (17-week-old). The mice were used an insulin syringe under 2.5% isoflurane inhalation anesthesia after surgical exposure of spleen. After 3 weeks, luciferin emission imaging of isoflurane-anesthetized animals was measured by using the IVIS Spectrum (Caliper Life Sciences) after intraperitoneal injection of D-luciferin (150 mg; Promega, P1043) into the mice.

### 2.4. ALT/AST

The blood samples were collected from ND and DIO mice after intrasplenic injection. The spinning condition for blood specimen tube was 4000 rpm for 15 min. Serum levels of alanine aminotransferase (ALT) and aspartate aminotransferase (AST)were measured using a commercial kit (Nanjing Jian Cheng Bioengineering Institute, C009-2-1, C010-2-1).

### 2.5. RNA-Sequencing (RNA-Seq)

The RNA-seq samples were liver tissue from ND and DIO group after intrasplenic injection. Every liver tissue sample had both liver metastasis nodules and adjacent nontumor tissues. Total RNAs were isolated using the TRIzol reagent following the manufacturer's instructions and sent to Novogene Co., Ltd. for clustering and sequencing. RNA sequencing data were collected using Next Seq System Suite v2.2.0.

### 2.6. Cell Migration Assay

Transparent polyethylene terephthalate (PET) membrane of 8.0 *μ*m pore size (Falcon, 353097) was used in cell migration experiments. Equal number of the viable cells (20,000 cells) resuspended in 200 *μ*l serum-free medium were seeded onto the upper wells of the transwell chambers. The lower chamber was supplemented with 700 *μ*l medium containing different concentration of CX3CL1 recombinant protein or blocking antibody. 48 hours later, the upper cells were removed using cotton swabs and the migrated cells were fixed with 4% paraformaldehyde and stained with 0.1% crystal violet. The migrated cells were counted under a light microscope in six random fields. Human recombinant protein (Novoprotein, C461), human CX3CL1 chemokine domain antibody (Biotechne, AF365), mouse recombinant protein (Novoprotein, CI38), and mouse CX3CL1 chemokine domain antibody (Biotechne, AF472) were used in cell migration assay.

### 2.7. Immunohistochemistry

All tissue samples were fixed in formalin and embedded in paraffin and then cut into 4 *μ*m thick sections. Liver sections were staining with Oil-Red O solution (Sigma) or hematoxylin and eosin (H&E). The sections were detected with primary antibody: CX3CR1(1 : 200, Proteintech, 13885-1-AP), CX3CL1(1 : 200, ABclonal, A14198), and Cytokeratin19 (1 : 1000, Abcam, ab15463) overnight at 4°C. After being incubated with the suitable second antibody, the sections were treated with diaminobenzidine and counterstained with hematoxylin. All the sections were photographed with a microscope.

### 2.8. ELISA Analysis

Liver tissues were obtained from DIO and ND mice with intrasplenic injection. The liver tissues were lysed with RIPA and cell lysate supernatants were collected by centrifugation immediately. The ELISA kits for CX3CL1 were purchased from Panchao Biotechnology (Shanghai, China) and used according to the manufacturer's instructions.

### 2.9. Western Blotting

The cells and liver tissues were lysed with RIPA containing completed protease and phosphatase inhibitor, collected for centrifugation. The protein concentration was measured by a BCA Protein Assay Kit (Pierce Biotechnology, Waltham, MA). After denaturation with loading buffer, the cell lysates were separated with 10%-15% sodium dodecyl sulfate (SDS) –polyacrylamide gel electrophoresis (PAGE) gel electrophoresis and transferred to a nitrocellulose membrane. The membrane was blocked with 5% (*m*/*v*) skim milk and incubated overnight at 4°C with primary antibodies: CX3CR1(1 : 1000, Proteintech,13885-1-AP), CX3CL1 (1 : 1000, Proteintech, 10108-2-AP), and GAPDH (1 : 10000, Abways, AB2000). Then, the membrane was washed 3 times in TBST (10 min. each time) at room temperature and incubated with secondary antibodies: goat-anti-mouse (1 : 10000, Abways, AB0102) and goat-anti-rabbit (1 : 10000, Abways, AB0101) at room temperature for 1 hour. Finally, the membrane exposure was performed using enhanced chemiluminescence (ECL) by the Bio-Rad system.

### 2.10. Real-Time PCR

Total RNA from cells was extracted using the TRIzol reagent (Takara) according to the manufacturer's instructions. Reverse transcription reactions were performed with PrimeScript RT Reagent kit (Takara, Dalian, China). The RT-PCR was subsequently performed with SYBR Premix Ex Taq (Bimake) using a Via7 instrument (Applied Biosystems). 18s RNA was used as the reference gene for quantification, and fold changes were calculated via the relative quantification method (2^-*∆∆*CT^). The CX3CR1 primer sequences were forward: 5′ AGTGTCACCGACATTTACCTCC 3′, reverse: 5′ AAGGCGGTAGTGAATTTGCAC 3′; the CX3CL1 primer sequences were forward: 5′ GGAAAGGGGAAGTTGTAGGC 3′, reverse: 5′ AATCCAAGGGAGAGGTGAGC 3′; the 18s primer sequences were forward: 5′ AGTGTCACCGACATTTACCTCC 3′, reverse 5′ AAGGCGGTAGTGAATTTGCAC 3′.

### 2.11. Flow Cytometry Analysis

The PDAC human cell lines were resuspended in FACS buffer (2% fetal bovine serum in PBS). 5 × 10^5^ ~ 1 × 10^6^ cells were used for cell surface staining. The cells were incubated with CX3CR1 primary antibody (1 : 100, Proteintech,13885-1-AP) for 30 min in the dark at 4°C. Then, the cells were washed by adding 1 ml FACS buffer and centrifuged 1500 rpm, 5 min, RT. After the first step incubation and washing, the cells were treated with 50 *μ*l Alexa Fluor 488 anti-rabbit secondary antibody (1 : 500, Abcam) for 30 min in the dark at 4°C. The secondary antibody staining without primary antibody was used as negative control. Then, the cells were washed and resuspended in 100~200 *μ*l FACS buffer for flow cytometer analysis.

### 2.12. Statistical and Bioinformatics Analysis

Gene Expression Omnibus (GEO) dataset GSE71729 containing 145 primary PDAC, 46 adjacent pancreases, 25 liver metastases, and 27 adjacent livers were used to investigate the expression of CX3CL1 and CX3CR1. Renji GEO used in this study are available in the GEO database under accession code GSE151580. Survival rate was calculated by the Kaplan-Meier method. Kaplan-Meier survival curves was performed with the log-rank Mantel-Cox test. The data from TCGA and GTEx were normalized and log_2_-transformed. All statistics were performed using GraphPad Prism 9.0 and Microsoft Excel. Data were presented as the means ± standard deviation. After testing for normal distribution, statistical analysis was performed using a one-way ANOVA, a two-way ANOVA, and a two-tailed Student's *t*-test as appropriate for dataset. All experiments with cell lines were done in at least triplicate. Error bars in this study represent the mean ± SD, except for bioluminescent emission, whose error bars represent the mean ± SEM (ns, *P* > 0.05, ^∗^*P* < 0.05, ^∗∗^*P* < 0.01, ^∗∗∗^*P* < 0.001, and ^∗∗∗∗^*P* < 0.0001).

## 3. Results

### 3.1. DIO Caused Increase of Body Weight and Liver Dysfunction

**Figure 1:**
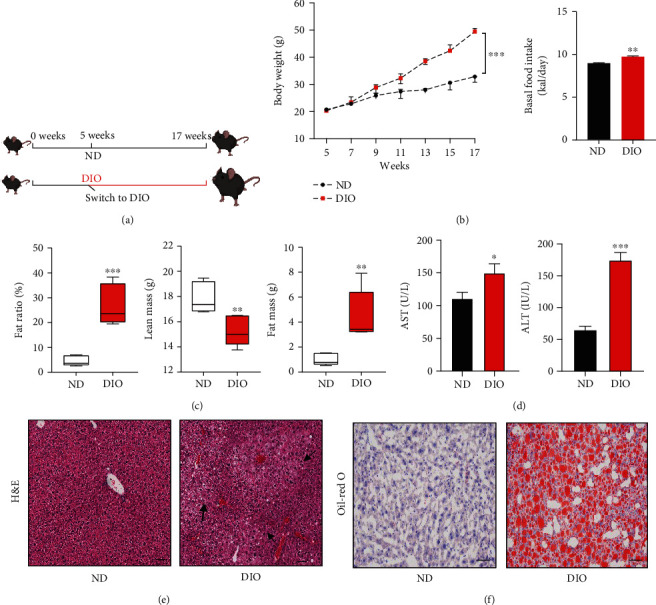
Establishment of a high-fat diet mouse model. (a) Overview of normal diet (ND) and diet-induced obesity (DIO) mouse model. The ND group and DIO group are all male mice. (b) Body weight and basal food intake of ND and DIO mice, *n* = 6/group. (c) Fat radio, lean mass, and fat mass of ND and DIO mice were measured using machine AccuFat-1050. (d) Serum chemistry of ALT and AST in ND and DIO mice. (e) Sections of ND and DIO liver tissue were stained with H&E, scale bar 100 *μ*m. Arrows, adipocyte enlargement. (f) Oil Red O staining was shown to present the lipid formation in livers from ND and DIO mice, respectively, scale bar 200 *μ*m.^∗^*P* < 0.05, ^∗∗^*P* < 0.01, and ^∗∗∗^*P* < 0.001.

To analyze the effects of DIO on liver metastasis of PDAC, we first performed a DIO model in C57BL/6J mice (Figure 1(a)). Mice fed a 12-week high-fat diet showed significantly increased body weight and basal food intake (Figure 1(b)). Mice gained approximately 2-fold weight on DIO mice than on ND mice. We next examined fat-associated differences by comparing DIO mice and age-matched ND mice. The results showed that DIO resulted in increased fat radio and fat mass, whereas lean mass was decreased by DIO (Figure 1(c)). In addition, the levels of serum alanine transaminase (ALT) and aspartate aminotransferase (AST) were elevated in DIO group compared to ND group (Figure 1(d)). Hematoxylin and eosin staining revealed that DIO caused obvious hepatic steatosis (Figure 1(e)). The number of fat accumulation were significantly increased in liver tissue of DIO mice compared to ND mice, revealed by Oil Red O staining (Figure 1(f)).

### 3.2. DIO Promoted Liver Metastasis of PDAC

**Figure 2:**
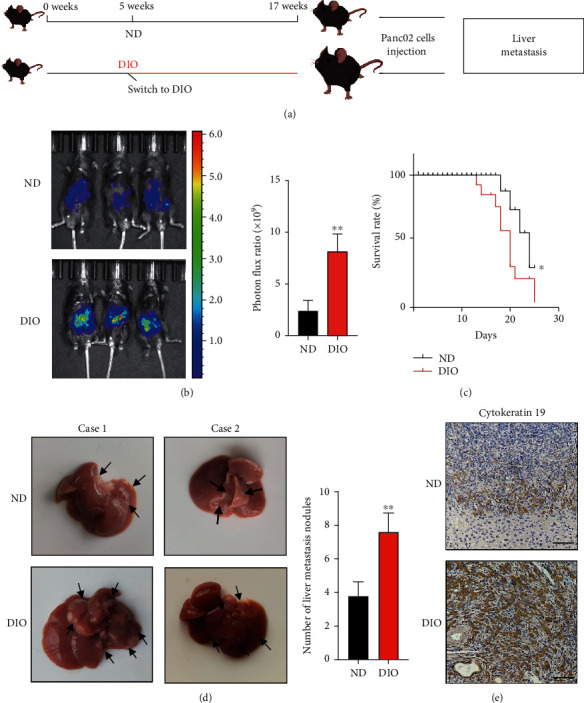
DIO promoted liver metastasis of PDAC. (a) Overview of liver metastasis mouse model. (b) Representative bioluminescence images of ND and DIO mice that injected luciferase-expressing Panc02 cells into spleen, *n* = 6/group, ^∗∗^*P* < 0.01. (c) Analysis of overall survival rate of ND and DIO mice, *n* =6/group. ^∗^*P* < 0.05. (d)Representative images of liver metastasis between ND and DIO. Black arrows, metastatic nodules. Relative number of liver metastasis nodules was quantified. (e) Representative IHC images of cytokeratin 19, scale bar, 50 *μ*m.

To explore the role of DIO in the liver metastasis of PDAC, we established a hepatic metastasis of PDAC by intrasplenic injecting the luciferase-labelled mouse PDAC cell line Panc02 into ND mice and DIO mice (Figure 2(a)). The growth of tumor cell in liver was monitored by in vivo bioluminescence imaging and expressed as luminescence intensity. In vivo imaging system revealed that an increased tumor burden in DIO mice compared with ND mice at 3 weeks. The data showed that the luminescence intensity of DIO mice increased approximately twice as compared to ND mice (Figure 2(b)). The survival rate was significantly decreased in DIO mice compared with ND mice (Figure 2(c)). We found that the number of liver metastatic nodules was significantly higher in DIO than in ND (Figure 2(d)). In addition, DIO had higher expression level of cytokeratin 19 compared with ND (Figure 2(e)). Taken together, our data suggested that DIO promoted liver metastasis of PDAC.

### 3.3. Chemokines Were Positively Correlated with DIO-Induced Liver Metastasis of PDAC

**Figure 3:**
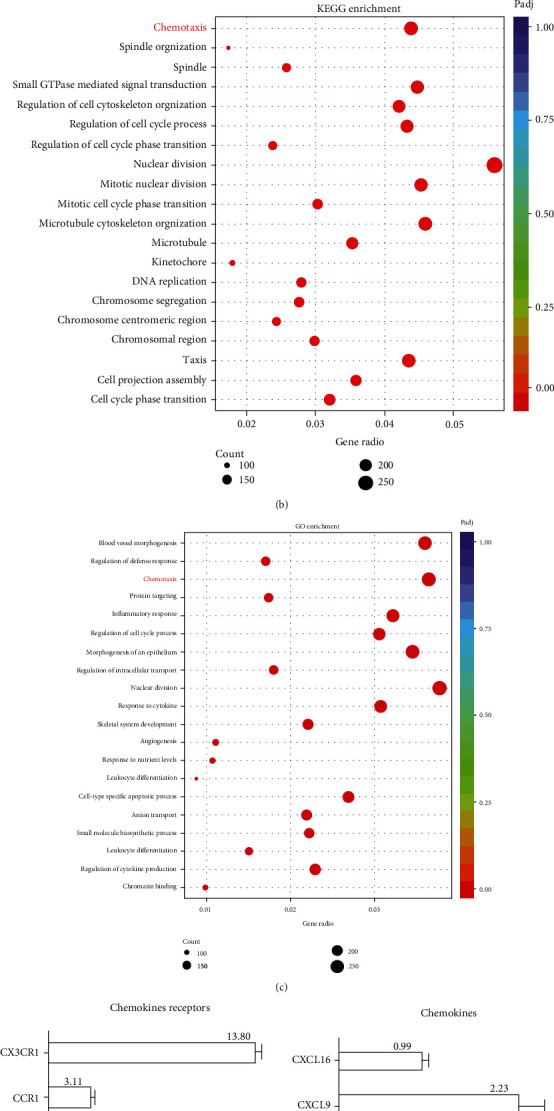
The expression of chemokines was positively correlated with DIO-induced liver metastasis of PDAC in mice. (a) Volcano plot of different gene expression in ND liver and DIO liver. Each point represented gene mRNA levels, fold change for ND and DIO. (∣Log_2_(FC) | ≦1.2, *P* value ≦ 0.05). (b, c) KEGG and GO pathway analysis on significantly upregulated genes between ND and DIO tissue samples. FDR < 0.01, Log_2_(FC) < ∣2∣. (d, e) Expression analysis of chemokines receptors and chemokines in livers; the expression of chemokines receptors and chemokines in DIO was normalized by ND. The multiples of the expression of chemokines and chemokines receptors in DIO were marked on the graph.

To further investigate the influence of DIO in liver metastasis of PDAC, we performed RNA-seq to analyze gene expression alterations in ND and DIO liver tissue after intrasplenic injection. The results indicated that total 4125 genes were differentially expressed, in which 2233 genes were upregulated and 1892 genes were downregulated in DIO group compared to ND group (Figure 3(a)). The KEGG and GO analyses of changed genes were performed to identify the biological process associated with liver metastasis, which revealed that chemotaxis-related pathways were extensively enriched in DIO compared to ND (Figures 3(b) and 3(c)). Based on biological pathway analyses of chemotaxis, we assessed the expression of upregulated chemotaxis-associated chemokines and chemokine receptors in liver of ND group and DIO group (Figures 3(d) and 3(e)). Interestingly, chemokine CX3CL1 and its unique receptor CX3CR1 had the same increased trend of expression levels in DIO liver compared with ND liver. These results suggest that the alterations of CX3CL1 and CX3CR1 may associate with important functions in DIO-induced liver metastasis of PDAC.

### 3.4. The Expression of CX3CL1/CX3CR1 Were Enhanced in Liver Metastasis of PDAC in DIO Mouse

**Figure 4:**
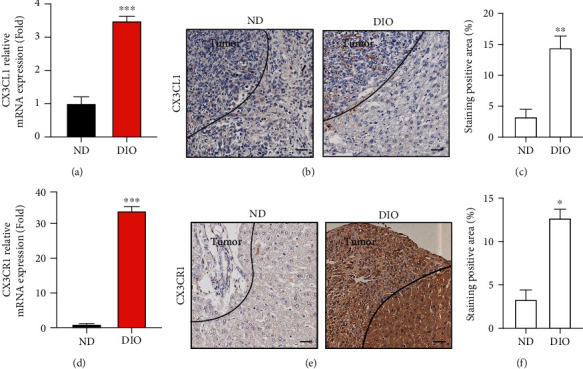
DIO increased expression level of CX3CL1 and CX3CR1 in liver metastasis of PDAC. (a) Relative mRNA levels of CX3CL1 in liver of ND mice and DIO mice after intrasplenic injection with 1 × 10^6^ Panc02 cells (*n* = 6/group). (b) Representative immunohistochemical images of CX3CL1 staining in liver of ND and DIO. Scale bar, 20 *μ*m. (c) Bar graph showed the relative CX3CL1 staining positive rate. (d) Relative mRNA levels of CX3CL1 in liver of ND mice and DIO mice after intrasplenic injection with 1 × 10^6^ Panc02 cells (*n* = 6/group). (e) IHC staining images of CX3CR1. Scale bar, 20 *μ*m. (f) Bar graph showed the relative CX3CR1 staining positive rate. ^∗^*P* < 0.05, ^∗∗^*P* < 0.01, and ^∗∗∗^*P* < 0.001.

Alteration of CX3CL1 and CX3CR1 was further confirmed by real-time PCR in liver tissue of ND and DIO. As a result, DIO increased mRNA levels of CX3CL1 and CX3CR1 (Figures 4(a) and 4(d)) compared with ND. We performed histochemical staining of liver tissue. The IHC images and relative positive staining indicated that CX3CL1 was more expressed on adjacent tumor tissue than liver metastasis nodules. DIO had higher CX3CL1 expression than ND in adjacent nontumor tissue (Figures 4(b) and 4(c)). The expression of CX3CR1 was highly expressed in DIO compared with ND in liver metastasis nodules (Figures 4(e) and 4(f)).

### 3.5. CX3CL1 Promoted Recruitment of CX3CR1-Positive Pancreatic Tumor Cells

**Figure 5:**
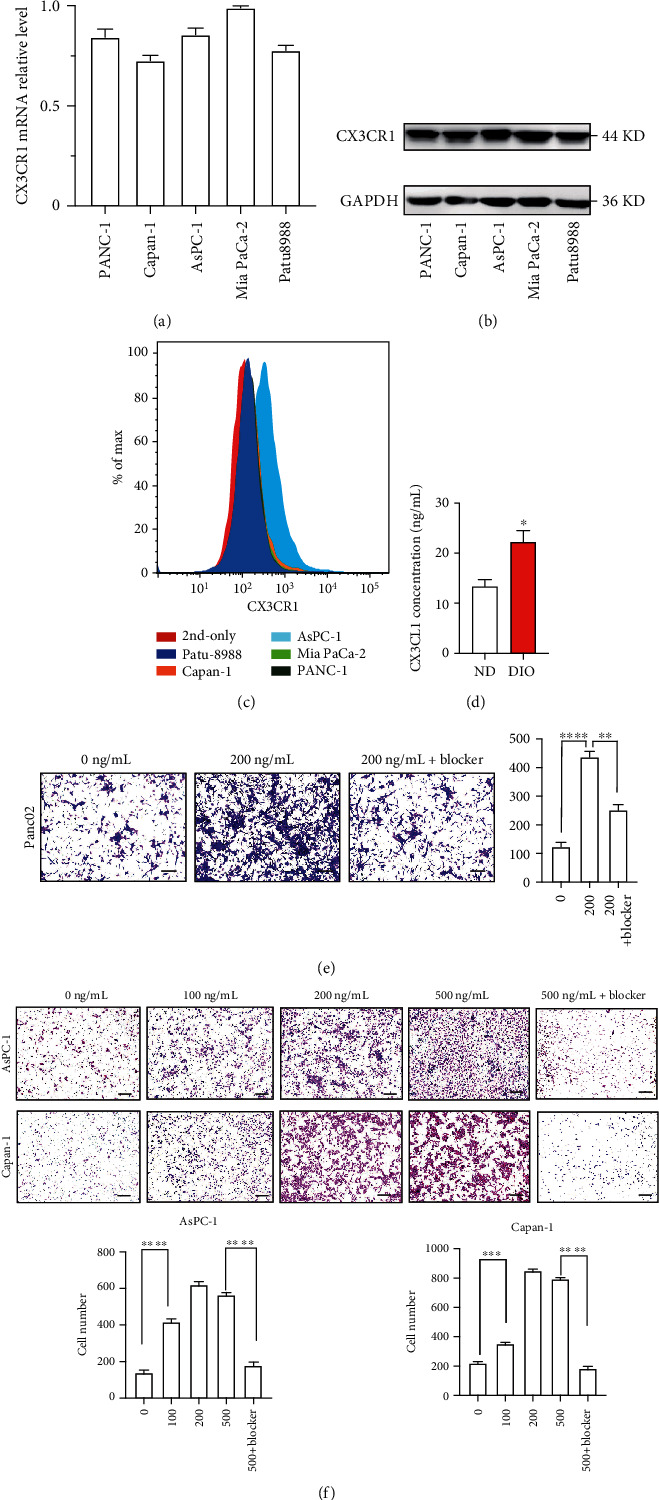
CX3CL1 promoted recruitment of CX3CR1-positive pancreatic tumor cells. (a) mRNA levels of CX3CR1 in PDAC cells. (b) Western blot analysis of CX3CR1 in human PDAC cell lines. (c) Flow cytometry analysis of the surface expression level of CX3CR1 in different human cell lines. (d)The expression level of soluble CX3CL1 was examined by using ELISA. Protein samples were obtained from the mouse liver. (e) Effect of CX3CL1 on the migratory ability of mouse PDAC cell lines Panc02; relative transferred cell numbers were analyzed. Cell migration experiments in blocker group were performed by using the blocking antibody of CX3CL1. The neutralization dose (ND_50_) is 1 *μ*g/ml in the presence of 30 ng/ml mouse recombinant CX3CL1. Scale bar 10 *μ*m. (f) Effect of CX3CL1 on the migratory ability of human PDAC cell lines AsPC-1 and Capan-1. Different concentration of CX3CL1 recombinant protein was used in cell transwell experiments; relative transferred cell numbers were analyzed. The neutralization dose (ND_50_) is 3 *μ*g/ml in the presence of 100 ng/ml human recombinant CX3CL1. Scale bar 10 *μ*m. ^∗^*P* < 0.05, ^∗∗^*P* < 0.01, ^∗∗∗^*P* < 0.001, and ^∗∗∗∗^*P* < 0.0001.

To analyze the mechanism by which CX3CL1 and CX3CR1 regulate liver metastasis, we first assessed CX3CR1 expression in tumor cell lines. The data of RT-PCR and western blot showed that CX3CR1 was highly expressed in 5 PDAC cell lines (Figures 5(a) and 5(b)). Flow cytometry was also performed to inspect the surface expression of CX3CR1 in PDAC cells. The results showed that CX3CR1 had the highest expression intensity in AsPC-1 (Figure 5(c)). In addition, we found that the expression level of soluble CX3CL1 was increased in the liver of DIO compared to ND (Figure 5(d)). Next, to determine the effect of increased CX3CL1 and its receptor, a series of migration assays of CX3CR1-positive cell lines were performed by using CX3CL1 recombinant protein and its blocking antibody *in vitro*. Mouse PDAC cell line Panc02 treated with recombinant CX3CL1 showed enhanced ability of migration compared with the control cell line. The migration of tumor cells was reduced in CX3CL1 inhibitor group (Figure 5(e)). Similarly, CX3CL1 markedly increased migration of PDAC cell lines AsPC-1 and Capan-1. This promotive effect was largely compromised by addition of neutralizing anti-CX3CL1 antibody (Figure 5(f)). These results suggest that CX3CL1 contributes to the recruitment of CX3CR1-positive tumor cells.

## 4. Discussion

DIO is associated with many types of tumors. DIO-induced inflammation accelerated prostate cancer growth via IL6 secreted by prostatic macrophages, as well phosphorylated STAT3- (pSTAT3-) positive tumor cells [23]. Studies have also shown that DIO induced tumor progression via adipose-secretory chemokines or cytokines within a xenograft mouse model of the prostate cancer cell line [24]. In addition, DIO regulated tissue stem and progenitor cell function. In previous reports, a robust peroxisome proliferator-activated receptor delta (PPAR-*δ*) enhanced stemness and tumorigenicity of intestinal progenitors by inducing DIO in intestinal stem cells and progenitor cells (nonintestinal stem cells) [25]. In the study of PDAC, DIO regulated the crosstalk between tumor-associated neutrophils (TANs) and pancreatic stellate cells (PSCs) that leads to PDAC growth [7]. The previous study has shown that DIO enhanced the development of a desmoplastic reaction, fibrosis, and inflammation in PDAC [7]. In our study, we found that several chemokines and chemokine receptors were upregulated in liver metastases of DIO. The relative tissue expression of the above-mentioned chemokines and chemokine receptors was also analyzed in primary PDAC by using TGCA and GTEx. The data showed that most of chemokines, such as CX3CL1, CXCL16, CXCL3, CXCL9, and CXCL5, were significantly high expressed in pancreatic tumor tissues compared to normal tissues (Figure S1A). A total of 6 chemokine receptors were upregulated in PDAC liver metastasis mouse model of DIO. The expression of these chemokine receptors was higher in pancreatic tumor tissues than normal tissues (Figure S1B). Meanwhile, we analyzed the correlation between the above-mentioned chemokines/chemokine receptors expression and patients' survival rate by using TCGA database. The expression of chemokines CXCL9 and CXCL5 had a significant correlation with PDAC patients' survival rate (Figure S3B). The above-mentioned 6 chemokine receptors expression have no statistical difference between PDAC patients' survival rate (Figure S3B).

In our study, we identified that DIO could promote liver metastasis of PDAC, and the expression of chemokines was altered in liver tissue of DIO mice compared to ND mice. In DIO mouse model, the data of RNA-seq showed that chemokine CX3CL1 and its unique receptor CX3CR1 played an important role in liver metastasis of PDAC. In addition, we also performed another mouse model of liver metastasis without DIO. We found that the mRNA levels of CX3CL1 and CX3CR1were increased in mice liver after injected with PDAC cells compared to normal liver (Figure S3A, B). The expression of CX3CL1 and CX3CR1 further increased along with the progression of liver metastasis, which indicates that the alteration of CX3CR1 and CX3CL1 in liver metastases may be a general phenomenon.

However, expression of CX3CL1 and CX3CR1 in clinical patient samples was opposite to that in mouse models. Analysis of CX3CR1 in clinical samples showed that the expression of CX3CR1 was higher in normal liver compared to metastatic liver (Figure S3C, D). Bioinformatic analyses were performed using a Gene Expression Omnibus (GEO) dataset GSE71729.The data showed that the alteration of CX3CL1 expression represented no statistical difference between normal liver and metastatic liver (Figure S3E). In dataset of Renji GEO, the level of CX3CL1 expression in normal liver was increased compared to metastatic liver (Figure S3F). The number of clinical samples were small, which maybe the reason for the different expression of CX3CL1 in two databases. In addition, how much tumor tissue and adjacent nontumor tissue was included in a liver sample could affect the cell types detected. The liver is a central immunological organ consisting of parenchymal cells (e.g., hepatocytes and cholangiocytes), nonparenchymal cells (e.g., Kupffer cells, hepatic stellate cells, and endothelial cells), and manifold cell types from adaptive and innate immune system. These cells were involved in balancing immunity and tolerance against pathogens [26]. Previously, several reports have demonstrated that CX3CL1 was expressed in several cell types. The main cellular source of CX3CL1 is hepatic stellate cells in the liver [27]. Therefore, the different regions of liver tissue maybe another reason for the difference in the analysis results of the two databases.

Our investigation focused on CX3CL1 and its unique receptor CX3CR1.Their detailed function in PDAC liver metastasis of DIO needs to be further explored. Firstly, the specific cell type of CX3CL1 has not been confirmed under DIO-fed in our study. This issue requires to extract various types of cells such as primary hepatocytes, hepatic stellate cells, tumor cells, and macrophages from mice. In addition, the functions of different forms of CX3CL1 have not been identified in liver metastasis of PDAC. In the previous reports, the expression level of CX3CL1 was regulated by inflammatory stimuli such as IL-1, TNF, and IFN-𝛄 [28]. CX3CL1 cellular form was regulated by TNF, which activated specific metalloproteases that cleave CX3CL1 from the plasma membrane [29]. CX3CL1 was associated with tumor cell invasion [30–32]. Usually, soluble CX3CL1 played a more important role in recruitment. In our study, we determined that soluble CX3CL1 was elevated in PDAC liver metastases of DIO compared to ND. Importantly, we found that increased CX3CL1 expression level resulted in further recruitment of PDAC tumor cells. The migration of tumor cells was reduced in CX3CL1 blocking group. Whether the membrane-bound and soluble forms of CX3CL1 had different recruitment roles in PDAC liver metastasis of DIO needs to be further investigated in the future. Moreover, to verify the recruitment of CX3CL1 to the CX3CR1-expressing tumor cells in PDAC liver metastasis of PDAC, we performed the transwell experiments by using in vitro recombinant protein. To some extent, the in vitro experiments were not the best model to illustrate the function of CX3CL1. We will extract primary tumor cells from DIO mice for more detailed studies. Some previous evidences showed that CX3CL1 played an important role in the tumorigenesis of obesity-associated cancers [19]. For example, it has been identified that CX3CL1 was a key regulator of CD8^+^ T cells, and abundance of CX3CL1 in the visceral adipose tissue was strongly correlated with metainflammation marker [33].

CX3CL1 exclusive receptor CX3CR1 was expressed in different tumor such as breast, liver, prostate, and pancreatic cancer. Previous studies also showed that CX3CR1 expression was observed in Kupffer cells/macrophages, B cells, monocytes, lymphocytes, smooth muscle cells, and natural killer (NK) cells [34]. In our study, we demonstrated the role of CX3L1 in recruiting tumor cells expressing CX3CR1. It is unclear whether CX3CL1 could recruit monocytes expressing CX3CR1 to the liver to further promote liver metastasis of PDAC in DIO. However, there are many contrasting results on the effect of metastasis ability of CX3CR1. For example, previous study demonstrated that overexpression of CX3CR1 was an early event in pancreatic cancer progression and regulated tumor invasion [35]. The CX3CR1-positive tumor cells could adhere to nearby infiltrating ganglia and neural cells expressing CX3CL1, which were preferred to local rather than distant tumor in human PDAC [17]. In contrast, other studies reported about prostate and breast cancer showed that the CX3CR1-positive tumor cells were circulating in the blood, attracted at distant sites by some ligand-expressing cells [30]. Therefore, the function of various types of cells expressing CX3CR1 will provide new insights into PDAC liver metastasis of DIO. The CX3CR1-knockdown tumor cells or transgenic mice will be used in the intrasplenic injection model to explore metastasis ability of CX3CR1.

## 5. Conclusions

In conclusion, this study showed that the chemokine/chemokine receptor CX3CL1/CX3CR1 axis was an important determinant of liver metastasis of PDAC induced by DIO. In DIO mice, the process of liver metastasis was accelerated, which resulted in poorer prognosis and increased scale of liver nodules. The expression levels of CX3CL1 and CX3CR1 were significantly upregulated in DIO-induced liver metastasis of PDAC compared to ND. Increased CX3CL1 promoted the recruitment of pancreatic tumor cells expressing CX3CR1. The complex functionality presented that CX3CL1/CX3CR1 was a potent therapeutic target.

## Data Availability

The RNA-seq data generated in this study have been deposited in the Sequence Read Archive (SRA) repository under accession code PRJNA820159. All the remaining data used to support the findings of this study are available from the corresponding author upon request.

## Supplementary Materials

Supplementary Materials See Figure S1, S2, S3 in the supplementary material for the expression and survival rate analysis of chemokines and chemokine receptors in PDAC patients and liver tissue of mice.
