# Supplementary material for: Diet-Induced Obesity Promotes Liver Metastasis of Pancreatic Ductal Adenocarcinoma via CX3CL1/CX3CR1 Axis
Source: J Immunol Res. 2022 Apr 18;2022:5665964. doi: 10.1155/2022/5665964 (PMC9038430; doi:10.1155/2022/5665964)
Supplement: Supplementary Materials — See Figure S1, S2, S3 in the supplementary material for the expression and survival rate analysis of chemokines and chemokine receptors in PDAC patients and liver tissue of mice. [file 5665964.f1.docx]

**Supplementary materials**

**Figure S1.** The expression of chemokines and chemokine receptors were altered in pancreatic tumor tissues compared to normal tissues. (A) Expression level of several chemokines in human pancreatic normal tissues and tumor tissues. Data combined with TCGA and GTEx database. ∣Log2(FC)∣≧1, ^*^P<0.01. (B) Expression level of several chemokine receptors in human pancreatic normal tissues and tumor tissues. Data combined with TCGA and GTEx database. ∣Log2(FC)∣≧1, ^*^P<0.01.


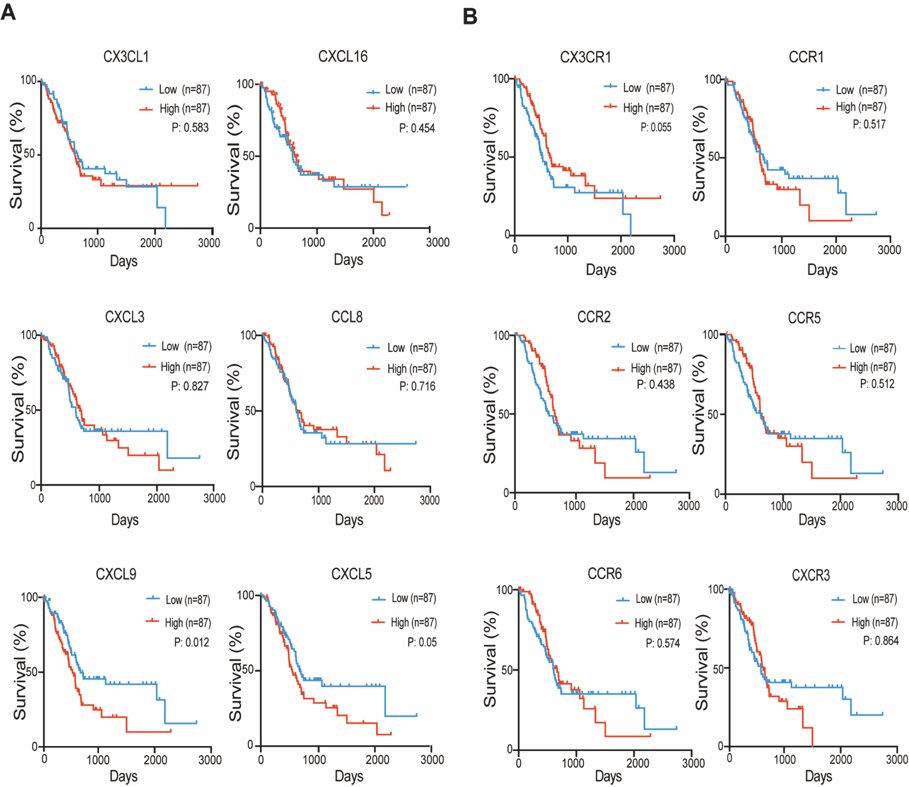


**Figure S2.** The analysis of chemokines and chemokine receptors on survival rate of PDAC patients. (A) The correlation between different chemokines expression and PDAC patients’ survival rate. High: high expression. Low: low expression. Data were obtained from TCGA database. (B) The correlation between different chemokine receptors expression and PDAC patients’ survival rate. High: high expression. Low: low expression. Data were obtained from TCGA database.


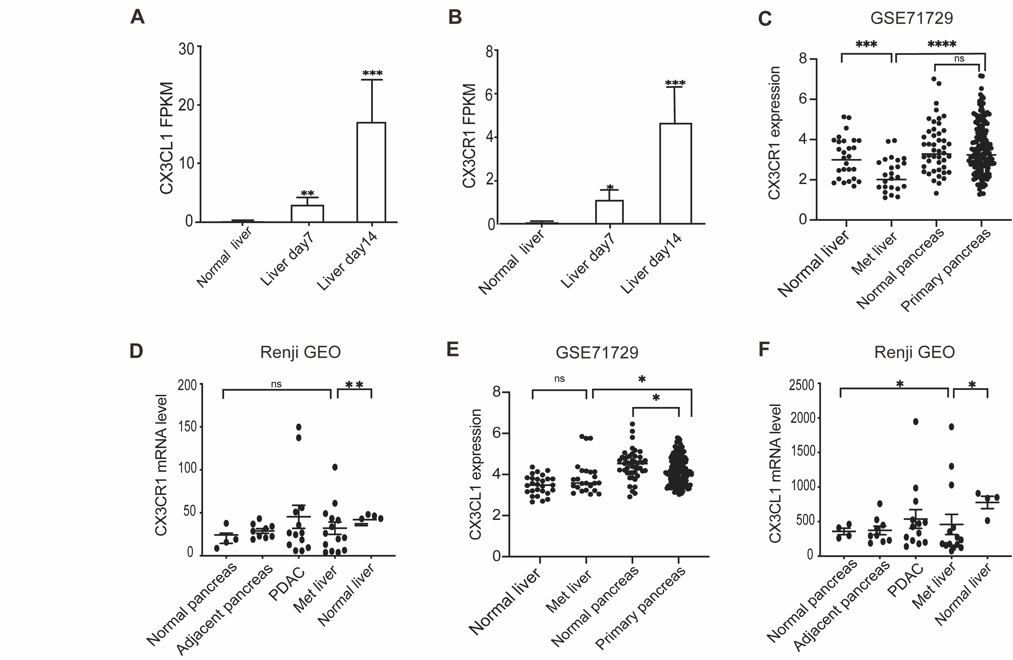


**Figure S3**. The alteration of CX3CL1 and CX3CR1expression in liver metastasis of PDAC mouse model and patients. (A, B) The alteration of relative CX3CL1 and CX3CR1 mRNA expression in liver metastasis of PDAC mouse model. Data from liver metastasis of PDAC in 7-week-C57BL/6J mice. (C, D) mRNA expression analyses of CX3CR1 in the normal pancreas, adjacent pancreas, primary PDAC, normal liver tissue and metastatic tissue. PDAC samples from the GSE71729 and Renji GEO database. (E, F) Expression levels of CX3CL1 in normal liver, metastasis liver, normal pancreas, primary pancreas from GSE71729 and Renji GEO database. ^*^P<0.05, ^**^P< 0.01, ^***^P < 0.001, ^****^P < 0.0001.
